# Proteins in the Cocoon of Silkworm Inhibit the Growth of *Beauveria bassiana*

**DOI:** 10.1371/journal.pone.0151764

**Published:** 2016-03-31

**Authors:** Xiaomeng Guo, Zhaoming Dong, Yan Zhang, Youshan Li, Huawei Liu, Qingyou Xia, Ping Zhao

**Affiliations:** State Key Laboratory of Silkworm Genome Biology, Southwest University, Chongqing, China; Institute of Plant Physiology and Ecology, CHINA

## Abstract

Silk cocoons are composed of fiber proteins (fibroins) and adhesive glue proteins (sericins), which provide a physical barrier to protect the inside pupa. Moreover, other proteins were identified in the cocoon silk, many of which are immune related proteins. In this study, we extracted proteins from the silkworm cocoon by Tris-HCl buffer (pH7.5), and found that they had a strong inhibitory activity against fungal proteases and they had higher abundance in the outer cocoon layers than in the inner cocoon layers. Moreover, we found that extracted cocoon proteins can inhibit the germination of *Beauveria bassiana* spores. Consistent with the distribution of protease inhibitors, we found that proteins from the outer cocoon layers showed better inhibitory effects against *B*. *bassiana* spores than proteins from the inner layers. Liquid chromatography-tandem mass spectrometry was used to reveal the extracted components in the scaffold silk, the outermost cocoon layer. A total of 129 proteins were identified, 30 of which were annotated as protease inhibitors. Protease inhibitors accounted for 89.1% in abundance among extracted proteins. These protease inhibitors have many intramolecular disulfide bonds to maintain their stable structure, and remained active after being boiled. This study added a new understanding to the antimicrobial function of the cocoon.

## Introduction

The silkworm cocoon has been well studied as the silkworm is the model lepidopteran insect [1–6], and its cocoon has important economic value. An early study revealed that *Bombyx mori* cocoon is mainly composed of fibroins and sericins [7], which have prominent physical properties to protect pupae [8]. Furthermore, some proteins with small molecular weight were found in the cocoon, including two protease inhibitors and two seroins [9–10]. The expression of protease inhibitors changed after infection by bacteria, fungi or viruses [11], indicating that they are immunity related proteins. Furthermore, many protease inhibitors showed inhibitory activity against the fungal proteases, as well as the germination of *Beauveria bassiana* conidia [12–15]. The expression of seroins was up-regulated after infection with bacteria and virus [16–18]. Moreover, seroins were found showing inhibitory activity against the growth of bacteria and nucleopolyhedrovirus [18]. In addition, some other immunity related proteins were identified in the silk gland and silk in previous studies. For example, a 18 wheeler protein was identified in silk, which was speculated to have antimicrobial effects [19]. The hemolin was found to have expression in the silk gland and function as opsonin in response to bacterial challenge [20].

By using liquid chromatography–tandem mass spectrometry (LC-MS/MS), Dong et al. (2013) identified hundreds of proteins in seven kinds of silk fibers spun by silkworm larvae at different developmental stages [21]. Besides protease inhibitors and seroins, some other antimicrobial components were identified in the silk. The presence of antioxidant enzymes, such as peroxidase, thioredoxin, and superoxide dismutase in the silk suggested that reactive oxygen species (ROS) may be generated during spinning, which has important roles in immune responses [22].

Fungi have potential abilities to destruct the cocoon by secreting proteases. To reveal the resistant function of cocoon proteins against the fungi, we extracted proteins from the cocoon by Tris-HCl buffer, and then determined their impact on the fungal growth. A fungal protease was used as the target enzyme to measure the activities of protease inhibitors in the cocoon. Furthermore, LC-MS/MS was used to identify the extracted cocoon proteins.

## Materials and Methods

### Materials

*B*. *mori*, (strain p50 (DaZao) and *B*. *bassiana* were provided by the State Key Laboratory of Silkworm Genome Biology, Southwest University, China. The silkworms were reared on mulberry leaves at a stable temperature of 25°C. Cocoon silk was collected and stored at 4°C until used. The fungus was cultured on potato dextrose agar (PDA) medium at 25°C and harvested after 2 weeks.

### Extraction and heat treatment of proteins from the cocoon

The cocoon was divided into six layers and then was cut into small fragments. The corresponding layers from four cocoons were collected as one group and then were weighted. Proteins were extracted from cocoon with 3 mL of 100 mM Tris-HCl buffer (pH 7.5) [19] for 30 min in a rotary shaker at 220 rpm at 37°C. After incubation, the extracts were centrifuged at 12,500 *g* for 10 min, at 4°C. The precipitates were collected and dried in room temperature and then were weighted. The supernatant was filtered through a 0.22 μm Millex-GP membrane (EMD Millipore, USA).

### Heat treatment of proteins from the cocoon

Proteins from different cocoon layers (extracted according to section 2.2) were boiled for 10 min and cooled on ice. Then, they were centrifuged at 14,500 *g* for 10 min, at 4°C. The supernatant and precipitate were then collected separately.

### Visualization of protease inhibitory activity in the cocoon

The activities of protease inhibitors were visualized by the method of Uriel and Berges [23], with a slight modification. Equal amounts (5 μg) of proteins (extracted according to section 2.2) were separated by the native polyacrylamide gel electrophoresis (PAGE). After electrophoresis, the gels were incubated at 37°C for 20 min with proteinase K solutions (0.07 mg/mL, Sigma–Aldrich, USA) in 0.1 M phosphate buffer, pH 7.4. Specific inhibitory activity toward proteinase K was visualized by staining the gels with the Fast Blue B Salt (Sigma–Aldrich) solution containing N-acetyl-dl-phenylalanine-b-naphthyl ester (Sigma–Aldrich).

### Activity assay of protease inhibitors from the cocoon

Protease inhibition assays were performed according to the previously reported method with a slight modification [24]. One microgram proteinase K was pre-incubated with 10 μg proteins from different cocoon layers (extracted according to section 2.2) in 100 μL of Fluoro^™^ Assay Buffer (100 mM Tris-HCl, pH 7.5) (G-Biosciences, USA) for 30 min at 37°C. FITC-casein substrate (G-Biosciences) in 100 μL of Fluoro^™^ Assay Buffer was then added and incubated at 37°C in the dark for 60 min. Substrate hydrolysis was monitored by measuring the excitation at 485 nm and emission at 535 nm in 96-well, fluorometer-compatible titer plates. The activity of protease inhibitors = 100%—(residual enzyme activity/original enzyme activity) × 100%

### Preparation of *B*. *bassiana* spores

Two-week-old conidia from *B*. *bassiana* grown on PDA medium were suspended in a 5 ml solution of 0.05% Tween-80 by vortexing for 2 min. Mycelia were removed by two rounds of filtration through a sterile muslin cloth. After filtration, the filtrate was centrifuged at 6000 *g* for 10 min at 4°C. The precipitate was washed in sterile Milli-Q double-distilled (dd) H_2_O and centrifuged at 6000 *g* for 10 min at 4°C. The supernatant was discarded and the spores were stored in sterile Milli-Q ddH_2_O.

### Inhibition assay of cocoon proteins against the growth of *B*. *bassiana*

A total of 100 μL of potato dextrose broth medium was blended with 65 μg protein from each cocoon layer (extracted according to section 2.2). Each of the blended media were inoculated with 2.5 × 10^6^ conidial suspensions. The control group was also composed of 100 μL PDB medium, 2.5 × 10^6^ conidial and Tris-HCl buffer. The total volume of all groups is 200 μL. All groups were incubated for 16 h at 26°C. The percentage of germinated conidia was assessed by hemocytometer measurement. All experiments were repeated three times. A conidium was considered to have germinated if the length of its germ tube was not smaller than its width. The germination rates among different groups were compared by one-way analysis of variance (ANOVA).

### Mass spectrometry of proteins extracted from the cocoon

The scaffold silk proteins, supernatant and precipitates of boiled scaffold silk proteins were prepared according to section 2.2 and 2.3. The precipitate of boiled scaffold silk proteins was dissolved in the 8M urea and the supernatant was in the 100 mM Tris-HCl buffer (pH 7.5). All these solutions were washed three times with 8 M urea using centrifugation at 12,000 g, 4°C for 20 min, reduced with 15 mM dithiothreitol for 120 min at 37°C and alkylated with 50 mM iodoacetamide for 60 min in the dark. Samples were washed three times with 8M urea and three times with 50 mM NH_4_HCO_3_.

The proteins were digested and identified according to the previous methods [25]. Briefly, proteins were digested by trypsin at a weight ratio of 1:50 (trypsin:protein) for 20 hours at 37°C. Tryptic peptides were recovered by centrifugation, lyophilized, and resuspended in 80 μL 0.1% formic acid. Tryptic peptides (2 μL) separated on a Thermo Fisher Scientific EASY-nLC 1000 system using a Thermo Fisher Scientific EASY-Spray column (C18, 2 μm, 100 Å, 50 μm × 15 cm) with a 140 min gradient of 2 min 3%~8% Buffer B (100% acetonitrile, 0.1% formic acid), 100 min 8%~20% Buffer B, 10 min 20%~30% Buffer B, 5 min 30% ~70% Buffer B, 3 min 70%~90% Buffer B, and 20 min 90% Buffer B. Peptides were analyzed using a Q Exactive mass spectrometer (Thermo Fisher Scientific, USA) in data-dependent mode with an automatic switch between MS and MS/MS scans using a top 20 method. Instrument parameters were: a resolution of 70,000 for a full MS scan and a 17,500 resolution for an MS2 scan, an automatic gain control target of 3e6 for a full scan and 1e6 for an MS2 scan, a maximum ion injection time of 20 ms for a full MS scan and 60 ms for an MS2 scan.

### Protein identification

Mass spectra raw data were analyzed using MaxQuant software (version 1.3.0.5) [26]. The MaxQuant searches were executed against an integrated silkworm proteome database from the National Center for Biotechnology Information (NCBI) and silkDB. Peptide searches were performed with the Andromeda search algorithm [27] using the following search parameters: a maximum of two missed cleavages permitted, carbamidomethyl cysteine as a fixed modification, and oxidation (methionine) and acetylation (of the N-termini of proteins) as variable modifications. Mass tolerance was 20 ppm for the first search and 6 ppm for the main search. The false discovery rate was 0.01 for both proteins and peptides, which had a minimum length of 6 amino acids. A minimum of one unique peptide was required for an identified protein. All common contaminants and reverse hits were removed. Identified peptides and proteins are shown in S1 and S2 Tables, respectively.

## Results

### Inhibitory activity of cocoon proteins against fungal proteinase K

**Fig 1:**
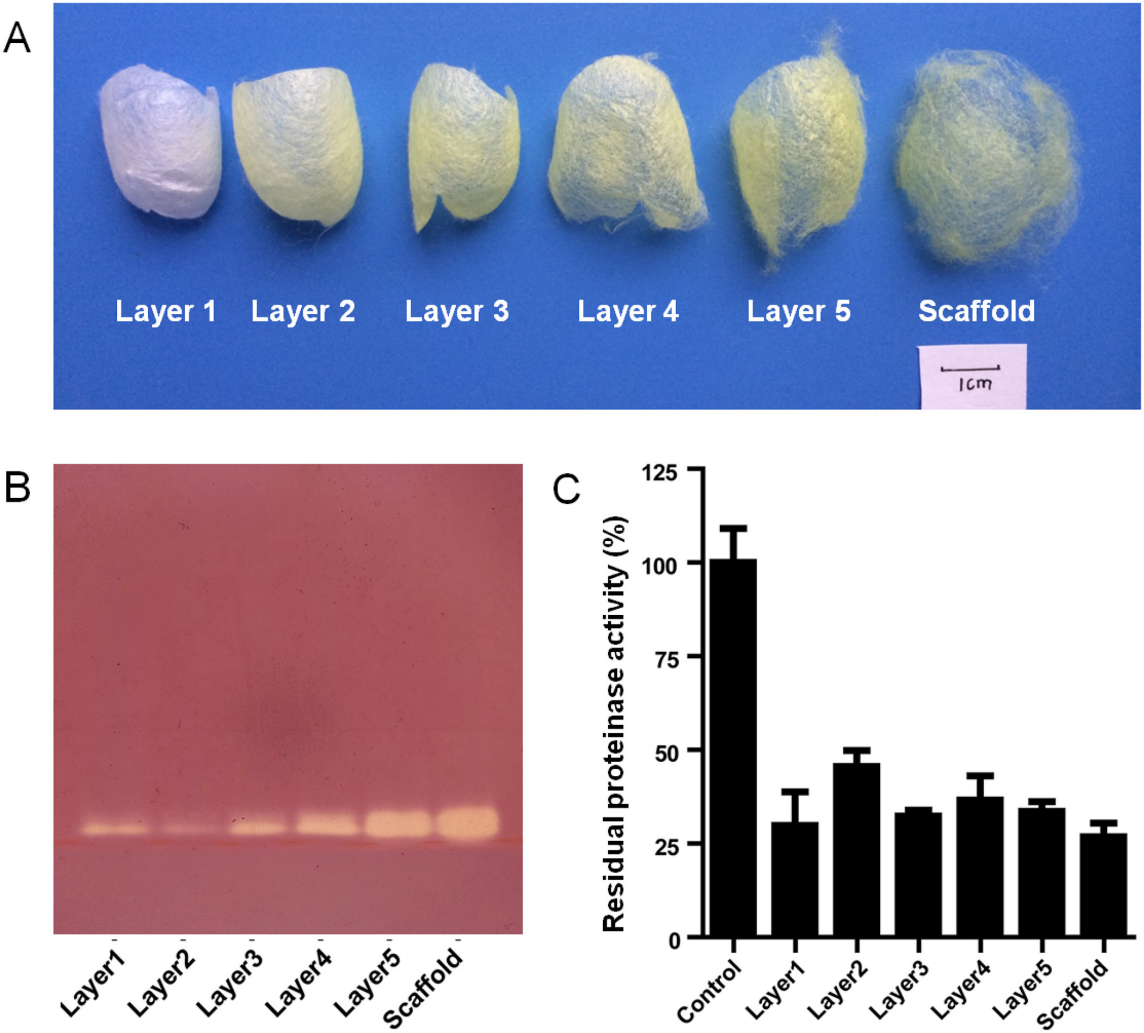
The activity and distribution of protease inhibitors in cocoon layers of *B*. *mori*. (A) Photos of different cocoon layers. From the inner layer toward the outer layer, the six cocoon layers were named as layers 1–5 (L1–L5) as well as the outermost scaffold. (B) Visualization of protease inhibitory activity of proteinase K inhibitors from different cocoon layers. Equal amounts (5 μg) of proteins from different cocoon layers were separated by native PAGE, and their inhibitory activity against proteinase K was visualized by staining the gels with the Fast Blue B Salt (Sigma–Aldrich) solution containing N-acetyl-dl-phenylalanine-b-naphthyl ester. (C) Inhibitory activity of cocoon proteins, isolated from different cocoon layers, against proteinase K. Equal amounts (10 μg) of proteins from different cocoon layers were used.

Cocoons were split into six layers, named as layers 1–5 from the inner to the outer layer, as well as the scaffold silk, the outermost layer (Fig 1A). Proteins were extracted from different cocoon layers according to section 2.2. The combination of results from three biological repeats indicated that only 4.08% proteins could be extracted from the inner cocoon layers, whereas 6.56% proteins were extracted from the outermost scaffold silk (Table 1).

**Table 1:** Extraction rate of cocoon proteins.

	Layer 1	Layer 2	Layer 3	Layer 4	Layer 5	Scaffold
Dry weight of cocoon layer* **(g)**	0.065±0.009	0.096±0.009	0.084±0.021	0.060±0.013	0.047±0.005	0.050± 0.010
**Dry weight of cocoon layer after extraction by Tris-HCl buffer (g)**	0.063±0.010	0.093±0.009	0.080±0.020	0.057±0.012	0.044±0.004	0.047± 0.009
**Extraction rate (%)**	4.08%±0.93%	3.91%±0.49%	4.41%±0.88%	5.05%±0.95%	5.86%±0.83%	6.56%± 1.04%

* Layers from four cocoons were collected into one tube as one experiment sample.

Active protease inhibitors in the extracts were visualized by incubating with the fungal proteinase K followed by staining with Fast Blue B Salt (Fig 1B). The amount of protease inhibitors tended to increase from the inner to the outer cocoon layers (Fig 1B). The proteins extracted from different cocoon layers showed distinct inhibitory activity against proteinase K (Fig 1C). The activity of proteinase K was reduced from 100% to 45.6% after being incubated with proteins from the layer 2, whereas the activity of proteinase K was decreased from 100% to 26.8% after being incubated with proteins from the scaffold silk (Fig 1C).

### Inhibitory activity of cocoon proteins against the germination of *B*. *bassiana* spores

**Fig 2:**
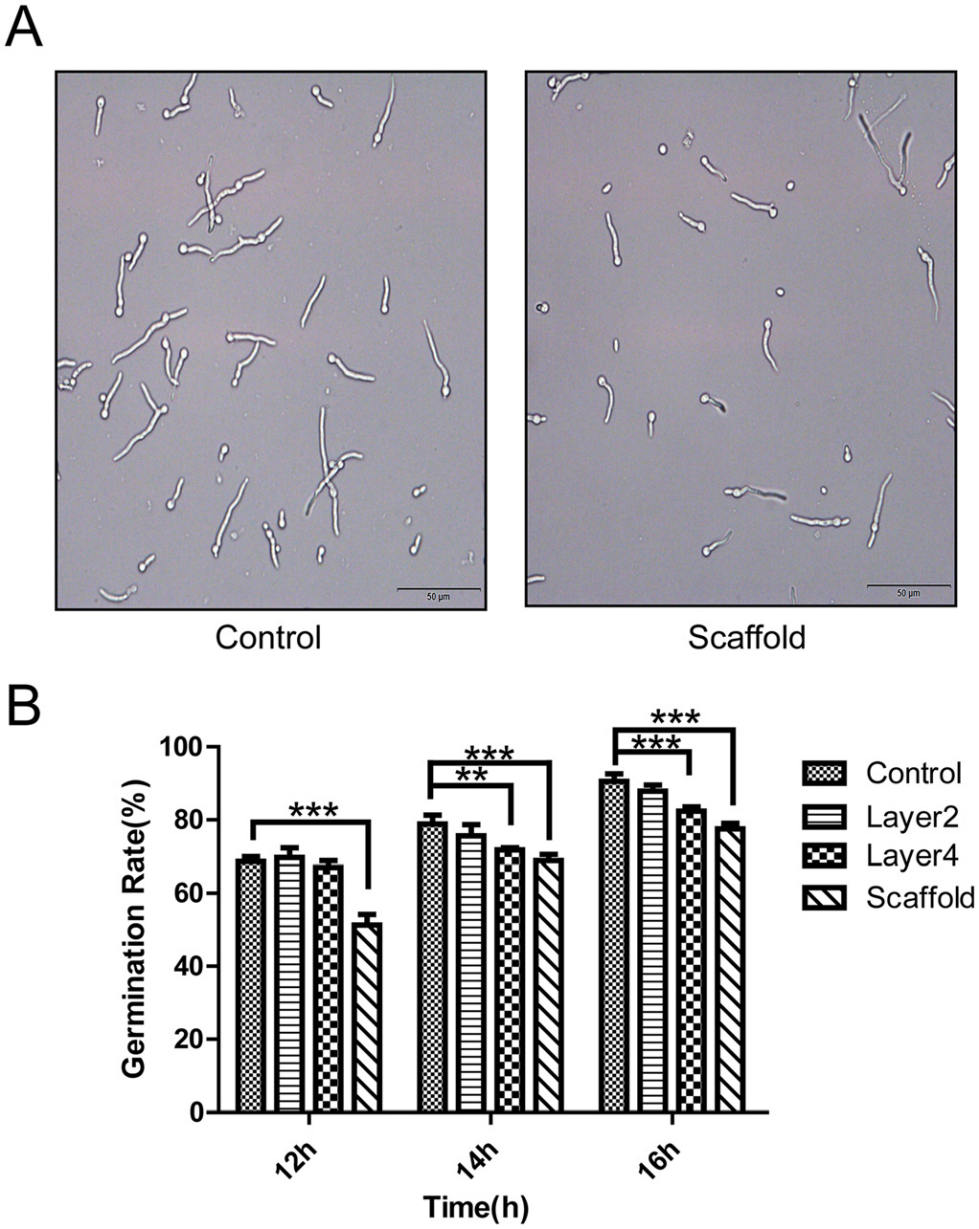
Inhibition of *B*. *Bassiana* conidia by proteins extracted from different cocoon layers. (A) Photographs showing the inhibition on germination of *B*. *bassiana* spores by the scaffold proteins. (B) The inhibition of *B*. *Bassiana* conidia by proteins from different cocoon layers. Equal amounts (65 μg) of proteins from different cocoon layers were incubated with the *B*. *Bassiana* conidia. ***P<0.001 and **P< 0.01 versus the control. Error bars indicate the standard error of the mean (n = 3).

Because the cocoon proteins could inhibit proteinase K secreted from fungi, we wanted to know whether cocoon proteins could inhibit the growth of *B*. *bassiana*, which is a major pathogenic fungus of silkworms. For the control experiment without cocoon proteins, most conidia germinated and the germ tube developed well (Fig 2A). When incubating the spores with cocoon proteins from different layers, we found that the germination of *B*. *bassiana* spores was obviously inhibited by cocoon proteins (Fig 2 and S3 Table). After 12 h of incubation, the germination percentages were 51.31% for scaffold silk, 67.44% for layer 4, 69.67% for layer 2, and 68.65% for the control. After 14 h of incubation, the germination percentages were 68.84% for the scaffold group, 71.78% for layer 4, 75.62% for layer 2, and 78.85% for the control. After 16 h of incubation, the germination percentages were 77.56% for the scaffold and 82.37% for layer 4. At all three time points, the scaffold (the outermost layer) proteins significantly (P<0.001) inhibited against spore growth (Fig 2). Cocoon proteins from the outer layers showed significantly stronger inhibition of spore germination than those from the inner layers (S3 Table). For example, proteins from the scaffold had significantly stronger inhibition than those from layer 2 after 12h (P<0.001), 14h (P<0.01), and 16 h (P<0.001) of incubation.

### Boiled cocoon proteins still have biological activity

**Fig 3:**
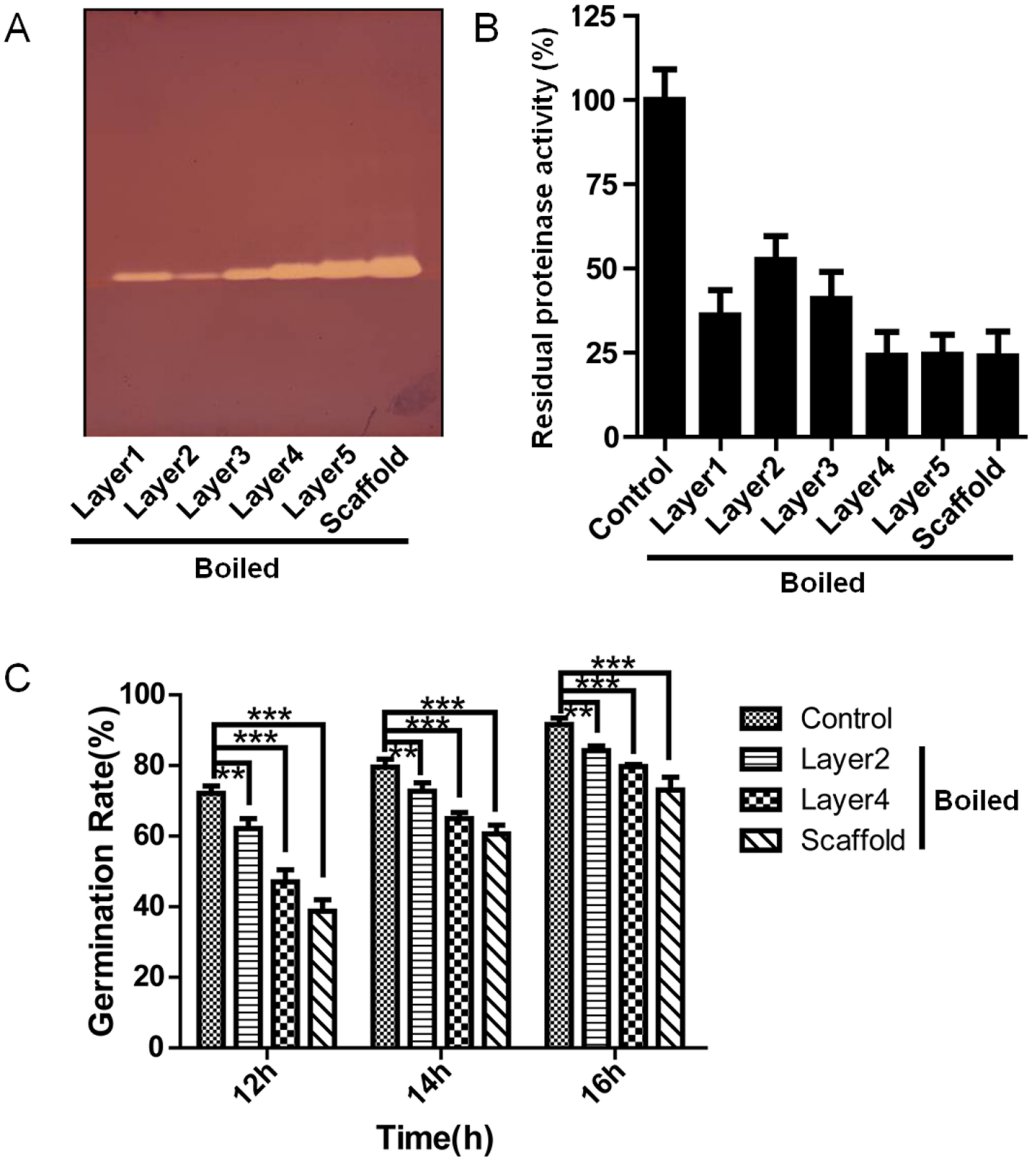
Inhibition of fungal proteinase K by boiled proteins extracted from different cocoon layers. (A) Visualization of protease inhibitory activity of proteinase K inhibitors from different cocoon layers. (B) Inhibitory activity of boiled proteins, isolated from different cocoon layers, against proteinase K. (C) The inhibition of *B*. *bassiana* conidia by boiled proteins isolated from different cocoon layers. Equal amounts of proteins, 5μg, 10μg, and 65μg, from different cocoon layers were used in (A), (B), and (C), respectively. ***P< 0.001 and **P< 0.01 versus the control. Error bars indicate the standard error of the mean (n = 3).

Proteins from different cocoon layers (extracted according to section 2.2) were boiled for 10 min, and then the supernatant were collected (prepared according to section 2.3). After being boiled, the active protease inhibitors in the supernatant were visualized by incubating with the fungal proteinase K followed by staining with the Fast Blue B Salt (Fig 3A). Consistent with the results in section 3.1, the boiled proteinase K inhibitors tend to increase in activity from the inner to the outer layers (Fig 3A). The boiled cocoon proteins could decrease the proteinase K activities by 47.4%-76.1% when compared with the control (Fig 3B). These results showed that boiled cocoon proteins still have inhibitory activities against proteinase K.

Like the non-boiled cocoon proteins, the boiled cocoon proteins could still inhibit the germination of *B*. *bassiana* spores (Fig 3C and S4 Table). After 12 h of incubation, the germination percentages were 38.69% for scaffold silk, 47.07% for layer 4, 62.09% for layer 2, and 72.09% for the control. After 14 h of incubation, the germination percentages were 60.58% for the scaffold group, 64.96% for layer 4, 72.71% for layer 2, and 79.54% for the control. After 16 h of incubation, the germination percentages were 73.01% for the scaffold, 79.74% for layer 4, 84.24% for layer 2, and 91.66% for the control. The layer 2 group significantly (P<0.01) inhibited spore growth at three time points, while the scaffold group and layer 4 group showed better inhibition (P<0.001) against spore growth than layer 2 group. The boiled cocoon proteins showed an increasing inhibition ratio against the *B*. *bassiana* spores from the outer to the inner cocoon layers, which was consistent to the changing trend of the proteinase K inhibitors. It is interesting that heat treatment increased the inhibition of spore germination by cocoon proteins when compared with the untreated cocoon proteins (S1 Fig). For the layer 2, layer 4 and scaffold after 12 h of incubation, the inhibition was increased by 7.6% (P<0.05), 20.0% (P<0.001), and 12.6% (P<0.01), respectively.

### Identification and quantification of extracted cocoon proteins

The above results showed that scaffold proteins had stronger inhibitory activity against *B*. *bassiana* spores when compared with proteins from other cocoon layers. Thus, we employed LC-MS/MS to investigate the Tris-HCl buffer extracted scaffold proteins (extracted according to section 2.2), as well as the supernatant and precipitate of boiled scaffold proteins (prepared according to section 2.3). In total, we identified 129 proteins, 30 of which were protease inhibitors (S2 Table).

**Fig 4:**
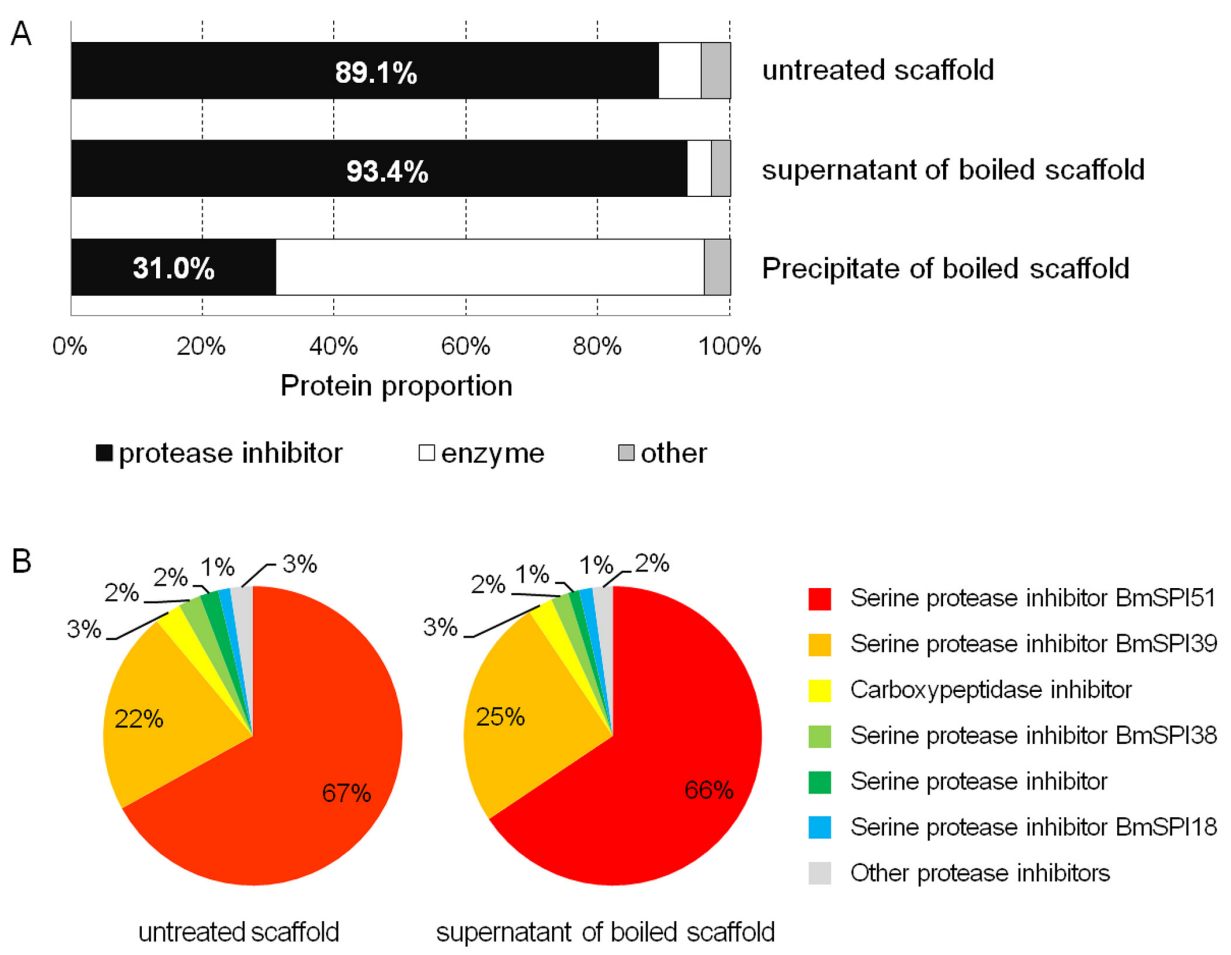
Identification and quantification of untreated scaffold proteins, the supernatant and precipitate of boiled scaffold proteins. (A) Relative abundance of proteins in different functional categories. (B) Relative abundance of protease inhibitors. The protein abundance was estimated by the intensity-based absolute quantification (iBAQ) algorithm in MaxQuant software.

The intensity-based absolute quantification (iBAQ) algorithm in MaxQuant software was used to estimate the relative protein abundance (Fig 4A and S2 Table). We found that protease inhibitors accounted for 89.1% of the total abundance of extracted scaffold proteins, while enzymes had a relative abundance of 6.5%, and the abundance of other proteins was only 4.4%. Regarding the supernatant of boiled scaffold proteins, the abundance of protease inhibitors increased to 93.4%, while the abundance of enzymes and other proteins decreased to 3.7% and 2.9%, respectively. For the precipitate of boiled scaffold proteins, enzymes accounted for 65.1%, while protease inhibitors only accounted for 31.0%. These results suggested that boiling led to the precipitation of many enzymes, and thus protease inhibitors showed an increase proportion after being boiled (Fig 4A).

Protease inhibitors in the untreated scaffold proteins and in the supernatant of boiled scaffold proteins were analyzed using a pie chart (Fig 4B). A Kunitz-type serine protease inhibitor BmSPI51 accounted for 67% and 66% of the total protein in the scaffold and boiled scaffold supernatant, respectively. The TIL-type serine protease inhibitor BmSPI39 had the second highest abundance, accounting for 22% and 25% of the total abundances in the scaffold and boiled scaffold supernatant, respectively. Other protease inhibitors only accounted for about 11% and 9% of the total proteins in the scaffold and boiled scaffold supernatant, respectively (Fig 4B and S2 Table).

## Discussion

The *B*. *mori* cocoon, which is composed of silk fibers, is a protective covering for the pupa. The cocoon has a multi-layer structure to protect the pupa with excellent mechanical properties. In this study, we divided the cocoon into six layers and extracted active proteins from the cocoon using Tris-HCl buffer. The extracted proteins could significantly inhibit the fungal protease K and the germination of *B*. *bassiana* spores. Futhermore, we found that protease inhibitors were the major components of extracted proteins, which showed a great stability under high temperature and their abundance tended to increase from the inner to the outer cocoon layers.

In insects, many signaling pathways have been reported to be involved in immune defense against pathogens, such as the melanization [28], Toll [29], immune deficiency (Imd) [30], and Jun-kinase (JNK) [31] pathways. Quinones [32], antimicrobial peptides [33] and lysozyme [34] are the final products of these pathways that directly kill pathogens or inhibit their growth. Many immune related components have been reported in the cocoon. However, their importance mainly depends on their amounts in the cocoon. To get active proteins from the cocoon, 100 mM Tris-HCl buffer (pH 7.5) was used to extract proteins at 37°C for 30 min. LC-MS/MS results suggested that only 0.04% fibroins and 0.34% sericins were extracted from the cocoon (S2 Table), while protease inhibitors accounted for 89.1% among extracted proteins. These protease inhibitors in the cocoon were found having inhibitory activities against the fungal proteinase and spore germination of *B*. *bassiana*.

The anti-fungal mechanism of protease inhibitors has been revealed in detail. Entomopathogenic fungi invade insects by the combination of mechanical pressure and enzymatic degradation [35–37]. The proteases secreted by the fungus are important virulence factors that are usually secreted at the time of conidial germination, and they participate in the penetrating process of the host cuticle [38–39]. Protease inhibitors of insects can inhibit the fungal growth by inhibiting their proteases [40]. In 1993, Eguchi et al. found that fungal protease inhibitor F in the silkworm blood could inhibit the proteases of *B*. *bassiana*, and prohibit spore germination of *B*. *bassiana* [41]. In 1997, Vilcinskas et al. purified some protease inhibitors from the blood of *Galleria mellonella* and found that they can delay the germination of *B*. *bassiana* spores [13]. In 2015, Li found that silkworm protease inhibitors BmSPI38 and BmSPI39 in the cuticle could inhibit the virulence protease CDEP-1 of *B*. *bassiana* as well as the germination of *B*. *bassiana* conidia [14]. In this study, we found that BmSPI38 and BmSPI39 accounted for 22% and 2% in the extracted protease inhibitors. BmSPI51 had the largest abundance and accounted for 67%, which was called a cocoon shell-associated trypsin inhibitor (CSTI) or BmSPI1 in previous studies. The inhibitory activity of BmSPI51 against trypsin has been demonstrated [42–44], but its activity towards microbial proteinases has not been tested.

Many protease inhibitors were reported to have good thermal and pH stability [42–43,45]. It is because that protease inhibitors contain many cysteine residues to form intramolecular disulfide bonds that could maintain stable structure. Among 30 protease inhibitors in the scaffold silk, 19 proteins have the TIL domain with 8–10 cysteines, 2 proteins have the kunitz domain with 6 cysteines, and 1 protein have the WAP domain with 8 cysteines [11]. These protease inhibitor have almost constant activities after being boiled. Moreover, heat treatment increased the inhibition of spore germination by cocoon proteins when compared with the untreated cocoon proteins. This may be because that some non-inhibitor proteins precipitated and were removed after boiling, which is equivalent to increasing the purities of protease inhibitors. Therefore, the protease inhibitors with stable structures and activities could provide lasting protection against fungi for the cocoon in the complex environment [21].

Lepidopteran larvae must go through the pupal stage to become adult moths or butterflies. Pupae cannot eat or move, but they face many natural enemies, including a variety of predators and pathogenic microorganisms. The cocoon is the best protective barrier for the pupa. In 2013, Chen et al. [8] found that the cocoon can provide complete protection for the pupa by its well-designed structure and properties. In this study, we found that cocoon protects pupa by antifungal proteins. On the basis of equal amounts of extracted proteins, the protease inhibitors in the cocoon showed a decreasing trend from the outer to the inner cocoon layers. In addition to the presence of more soluble proteins in the outermost layer (6.56%) than in the innermost layer (4.08%), the actual contents of protease inhibitors in the outermost layer should be more than that detected. The cocoon proteins in the outermost layer also showed the best inhibition against the germination of *B*. *bassiana* spores when compared with proteins from other layers. The outermost cocoon layer is directly exposed to pathogens. Thus, more protease inhibitors in the outermost layer could provide better protection for the cocoon. The inner layers of the cocoon have fewer protease inhibitors, but they have a denser silk distribution and better elastic modulus and tensile strength, which allow the cocoon to resist outside forces. It is the combination of the structure and composition of the cocoon that provide complete protection for the pupa inside, which allows the silkworm to safely complete its metamorphosis into a moth.

## Supporting Information

S1 Fig Comparison of the inhibitory effects of untreated cocoon proteins and boiled cocoon proteins on the germination rates of *B*. *Bassiana* conidia. ***P<0.001 and **P< 0.01 versus the untreated proteins. Error bars indicate the standard error of the mean (n = 3).
(TIF)

S1 Table Identified peptides from the scaffold silk of *B*. *mori*. Identified proteins are listed by the sequence, length, number of miscleavages, the charge states, the Andromeda score, mass, and the lowest posterior error probability (PEP) from multiple analyses. The PEP is a measure of the probability of a false hit being derived from the peptide’s score and its length.
(XLSX)

S2 Table Identified proteins from the scaffold silk of *B*. *mori*. Identified proteins are listed by functional category, accession number in the silkDB and GenBank databases, annotated name, gene ontology annotation, molecular weight, enzyme commission number, the number of peptides, and sequence coverage. The intensity-based absolute quantification (iBAQ) intensities are listed for each sample.
(XLSX)

S3 Table The germination rates of *B*. *bassiana* spores after being incubated with proteins from different cocoon layers. (XLSX)

S4 Table The germination rates of *B*. *bassiana* spores after being incubated with boiled proteins from different cocoon layers. (XLS)
