# Supplementary figures and images for: Proteins in the Cocoon of Silkworm Inhibit the Growth of Beauveria bassiana
Source: PLoS One. 2016 Mar 31;11(3):e0151764. doi: 10.1371/journal.pone.0151764 (PMC4816445; doi:10.1371/journal.pone.0151764)

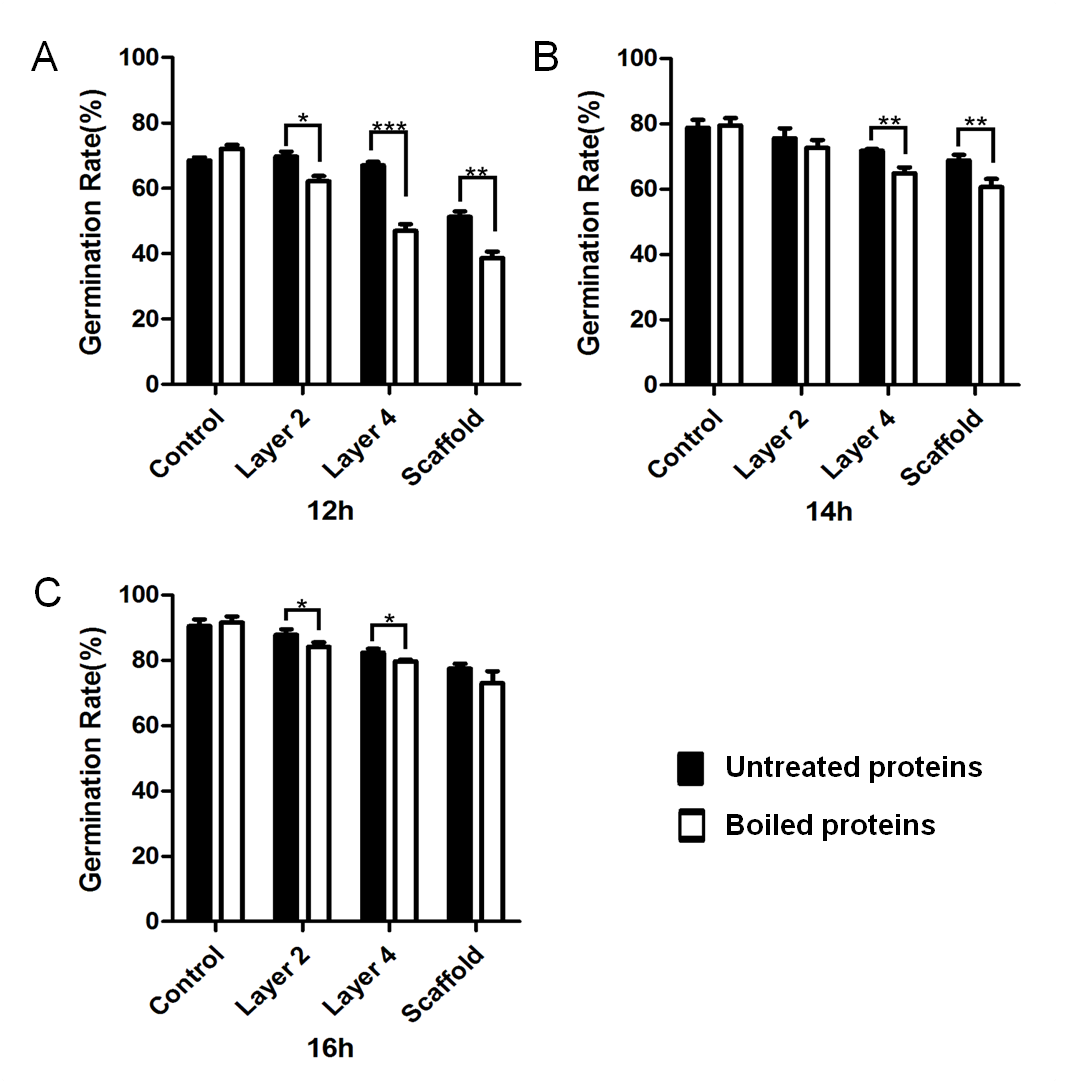

Supplement: S1 Fig — ***P<0.001 and **P< 0.01 versus the untreated proteins. Error bars indicate the standard error of the mean (n = 3). (TIF) [file pone.0151764.s001.tif]
